# Chimeric antigen receptor-T cell therapy for lung cancer: The tumor microenvironment bottleneck and remedies to circumvent it

**DOI:** 10.1016/j.pccm.2025.08.005

**Published:** 2025-09-13

**Authors:** Qi Zhou, Fengfei Sun, Xinhui Wang

**Affiliations:** a Department of Pulmonary and Critical Care Medicine, The Fifth Affiliated Hospital of Sun Yat-sen University, Zhuhai, Guangdong 519000, China; b Division of Gastrointestinal and Oncologic Surgery, Department of Surgery, Massachusetts General Hospital, Harvard Medical School, Boston, MA 02138, USA

Edited by: Peifang Wei

Lung cancer is the most lethal solid malignancy in adults worldwide.^1^ Chimeric antigen receptor (CAR)-T cell therapy has emerged as a promising approach for anti-tumor immunotherapy.^2^ As of November 2024, the United States Food and Drug Administration has granted market authorization to seven CAR-T products for treating relapsed or refractory hematological neoplasms; however, extending this platform to lung cancer faces formidable obstacles.^3^ Principal challenges encompass the unique lung tumor microenvironment (TME), inherent inter- and intratumoral heterogeneity, and T cell exhaustion. Realizing the full therapeutic potential of CAR-T cells in lung cancer requires urgent investigation and strategic resolution of these constraints. This article systematically appraises the current clinical landscape and offers a comprehensive framework to guide future CAR-T application in lung cancer.

CAR-T cells targeting a variety of immune cell surface antigens are effective therapies for certain hematologic malignancies.^4^ While it has made significant strides, particularly in targeting CD19 and B cell maturation antigen (BCMA), its current applications are primarily limited to B-cell acute lymphoblastic leukemia (B-ALL), B-cell lymphoma, and multiple myeloma.^5^ Compared with solid tumors, hematological tumors have lower antigenic heterogeneity; their well-defined and uniformly expressed antigens allow CAR-T cells to precisely target tumor cells and exert therapeutic effects. The expression levels of most tumor-specific antigens in solid tumors are often low and exhibit greater antigenic heterogeneity, increasing the likelihood of antigen escape. Solid tumors also possess a unique TME characterized by aberrant vasculature, immunosuppressive cells, and an extracellular matrix that supports immunosuppression. These features collectively hinder the infiltration of CAR-T cells into the TME.^6^ How these bottlenecks can be broken is the current focus of CAR-T cell therapy for solid tumors.

Tumor initiation and progression are intricately linked to T cell exhaustion. In CAR-T cell therapy, heightened levels of T cell exhaustion have been associated with diminished rates of treatment remission, leading to compromised effector T cell function. Recently, Wang et al^7^ conducted a comprehensive analysis of mouse hepatocellular carcinoma models, demonstrating that tumor cells highly expressing sulfotransferase 2B1 led to the inactivation of dedicator of cytokinesis protein 2 by releasing cholesterol sulfate, contributing to the depletion of invasive CD8^+^ cytotoxic T cells within tumors (Fig. 1A). T cell exhaustion is the key bottleneck that has hindered the application of CAR-T in solid tumors. In the future, through techniques such as gene editing and metabolic reprogramming, it is expected that the decline of T cell function can be significantly delayed, thereby enhancing the sustained killing ability and clinical remission rate of CAR-T in solid tumors.

**Fig. 1:**
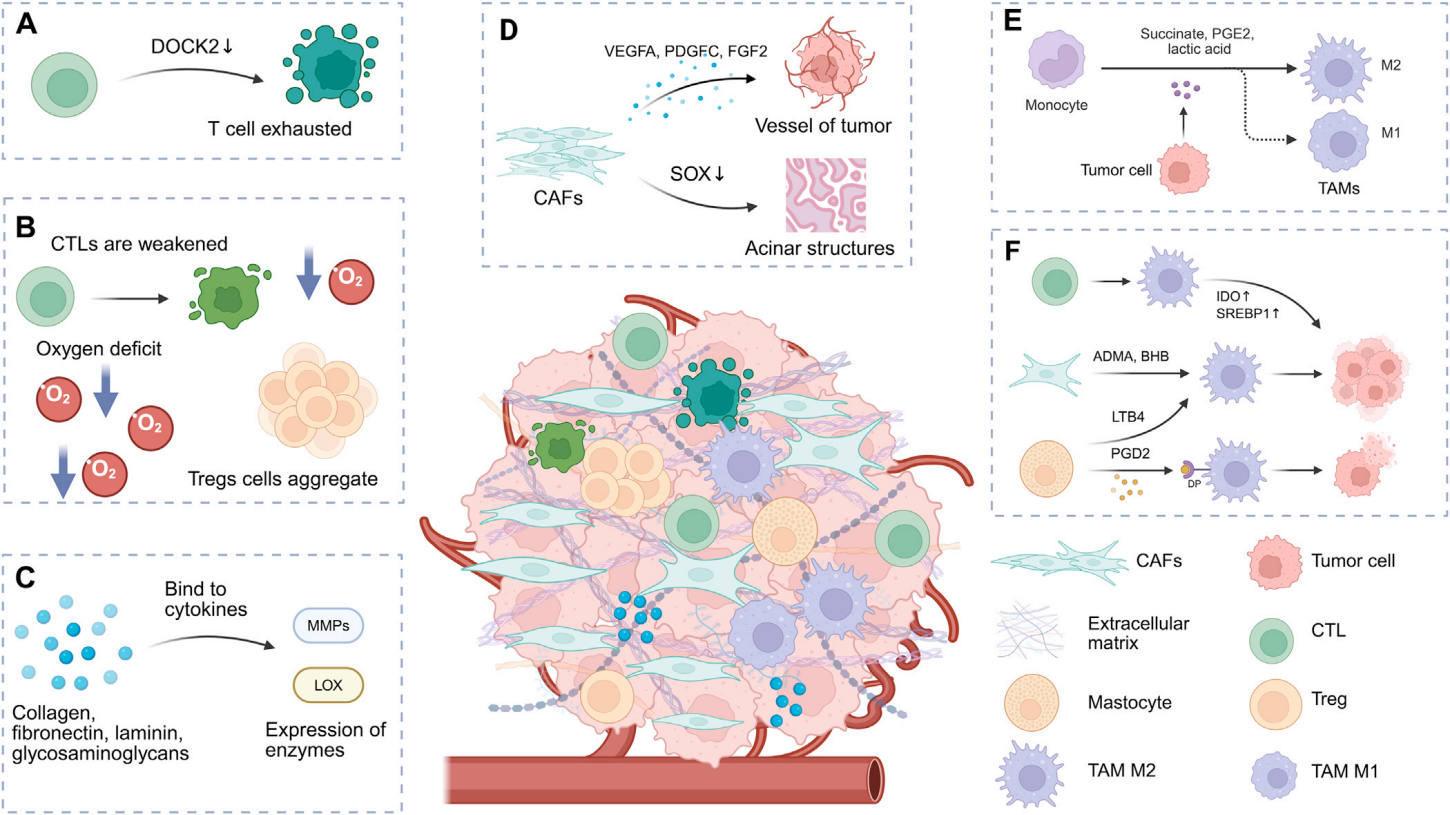
Important role of TME cells and related components. ADMA: Asymmetric dimethylarginine; BHB: β-hydroxybutyric acid; CAFs: Cancer-associated fibroblasts; CTLs: Cytotoxic T lymphocytes; DOCK2: Dedicator of cytokinesis 2; DP: PGD2 receptors; FGF2: Fibroblast growth factor 2; IDO: Indoleamine 2,3-dioxygenase; LOX: Lysyl oxidase; LTB4: Leukotriene B4; MMPs: Matrix metalloproteinases; PDGFC: Platelet-derived growth factor C; PGD2: Prostaglandin D2; PGE2: Prostaglandin E2; SOX: SRY-box transcription factor; SREBP1: Sterol regulatory element binding protein 1; TAM: Tumor-associated macrophage; TME: Tumor microenvironment; Tregs: Regulatory T cells; VEGFA: Vascular endothelial growth factor A. Created with BioRender.com.

Various T cell subtypes present in the TME play pivotal roles in either eradicating tumor cells or suppressing tumor immune responses, influencing TME metabolic dynamics. Regulatory T cells (Tregs) represent a subset of CD4^+^ T cells within the TME that exert immunosuppressive functions. By inhibiting the development of anti-tumor immunity, Tregs play a crucial role in impeding immune surveillance of cancer, hampering the generation of an effective anti-tumoral immune response within the host. Hypoxia is a prevalent feature of the TME and can impede cytotoxic T lymphocyte (CTL) function while attracting Tregs, diminishing tumor immunogenicity and promoting aggressive clonal expansion of heterogeneous tumor cells (Fig. 1B).^8^ Modulating transcription responsible for T cell differentiation and exhaustion represents a promising approach to enhancing the durability of the anti-tumor efficacy of CAR-T cells.

The TME comprises the vasculature, cytokines, and growth factors, along with diverse populations of stromal cells, which affect the function of tumor cells and the killing effect of the immune system.^9^^,^^10^ Lung cancer exhibits mutational heterogeneity, which extends beyond tumor epithelial cells to include the TME, whose immunosuppressive, angiogenic, and inflammatory properties facilitate cancer cell growth, invasion, and metastasis.^11^ Compared with other solid tumors, lung cancer exhibits a more profoundly immunosuppressive TME. Malignant cells actively secrete immunosuppressive mediators (transforming growth factor-β [TGF-β], interleukin-10 [IL-10]) while recruiting regulatory T cells and myeloid-derived suppressor cells to establish a potent immunosuppressive network. Inhibitory factors severely impair CAR-T cell activation and cytotoxic function, leading to therapeutic resistance.

The extracellular matrix (ECM) is one of the major components of the solid tumor TME, playing a crucial role in microenvironment regulation. It is often accompanied by the expression of remodeling enzymes, such as matrix metalloproteinases (MMPs) and lysyl oxidase. TME MMP expression levels can represent the degree of malignancy as they reflect their structural remodeling function in the progression of various epithelial cancers, including lung, breast, and pancreatic cancers (Fig. 1C).^12^ Therefore, to improve the efficacy of CAR-T cell therapy, future work must focus on developing co-targeting strategies that disrupt the ECM barrier (e.g., using MMP inhibitors) in combination with CAR-T cells, or on engineering CAR-T cells to resist the immunosuppressive effects of the remodeled matrix.

Cancer-associated fibroblasts (CAFs) are the most abundant stromal cell type in the TME and serve as the primary source of the ECM. CAFs produce a variety of pro-angiogenic factors, which contribute to angiogenesis in tumor tissues.^13^ CAFs are inversely correlated with prognosis in patients with non-small cell lung cancer (NSCLC).^14^ However, in certain contexts, CAFs exhibit a “double-edged sword” effect on tumor tissue. CAFs and ECMs can remodel intrinsic carcinogenic changes closely related to lung squamous carcinoma disease phenotypes, enhancing acinar structure formation by inhibiting SOX2 activity (a transcription factor associated with stemness) (Fig. 1D), thereby re-establishing the balance point between growth and differentiation,^15^ which may open new avenues for future anti-tumor strategies targeting CAFs. Due to the diversity of CAF origins, no specific biomarkers targeting CAFs have been developed. Even within the same tumor, it is challenging to identify CAFs using a single marker. Consequently, CAR-T cells capable of recognizing multiple CAF subpopulations in parallel are required to circumvent marker limitations and achieve targeted disruption of the tumor stroma.

Of the inflammatory cells in the TME, a significant amount of research focus has been placed on tumor-associated macrophage (TAMs) because of their association with poor prognosis and treatment resistance, including resistance to immunotherapy. TAMs exhibit dynamic phenotypic switching to adapt to their microenvironment; M1-like macrophages produce immunostimulatory cytokines that activate adaptive immune responses, while M2-like macrophages promote tissue repair processes and inhibit adaptive immune responses through the secretion of growth factors (Fig. 1E). Non-tumor cellular components within the TME can also engage in metabolic crosstalk with TAMs. Activated T cells can enhance indoleamine 2,3-dioxygenase (IDO) and sterol regulatory element-binding protein 1 (SREBP1) expression in TAMs, while components, such as asymmetric dimethylarginine (ADMA) and β-hydroxybutyrate (BHB) produced by CAFs, as well as leukotriene B4 (LTB4) released by mast cells, contribute to guiding TAMs in promoting tumorigenesis. Conversely, prostaglandin D2 (PGD2) from mast cells may bind to the PGD2 receptors (DP) on TAMs, potentially imparting antitumor effects (Fig 1F).^16^ Reducing the density or functional impact of TAMs can inhibit tumor growth, establishing them as a viable target for CAR-T cell therapy.

The application of CAR-T cell therapy to solid tumors, such as lung cancer, necessitates further optimization of existing CAR-T cell strategies. Genetic and non-genetic methods for reprogramming CAR-T cells have been developed in recent years and have been shown to enhance the anti-tumor efficacy of CAR-T cell therapy in pre-clinical studies. Wang et al^17^ developed a novel non-hereditary approach for reprogramming CAR-T cells by targeting cancer cells under stress; this non-genetic method involves exposing CAR-T cells to tumor cells subjected to cellular stress inducers, specifically disulfiram (DSF) and copper (Cu) (DSF/Cu), as well as ionizing radiation. This reprogramming enhances the cytotoxicity of CAR-T cells, which allows for more durable expansion *in vivo* while minimizing T cell depletion within the TME. Furthermore, in the same paper by Wang et al,^17^ it was indicated that stressed target cancer cells can also help reverse the immunosuppressive conditions of the TME. In the study utilizing reprogrammed B7-H3 CAR-T cells derived from patients with metastatic triple-negative breast cancer to treat patient-derived xenograft models, 100 % of mice achieved a complete response, which was associated with increased *in vivo* expansion of CAR-T cells and prolonged tumor-free survival. This study addressed many of the significant barriers currently limiting the efficacy of CAR-T cells against various solid tumors. Additionally, the persistent residual reprogrammed CAR-T cells demonstrated a long-term immunological memory response, with 60–100 % of mice remaining protected during the first tumor rechallenge and 60 % being protected during the second rechallenge. The stimulation of target cancer cells using DSF/Cu combined with ionizing radiation represents a promising strategy to enhance CAR-T therapy efficacy in solid tumors and to induce long-lasting immune anti-tumor memory responses.

Armored CAR-T cells have potentially enhanced functionality, as well as the potential to boost endogenous immune support and deliver tumor-killing agents directly, overcoming various challenges. By engineering CAR-T cells to modulate the cytokine milieu, it is feasible to activate the TME and immune cells, enhancing the anti-cancer capabilities of CAR-T and resident immune cells. Jaspers et al^18^ designed an anti-delta-like ligand 3 (DLL3) SC16 antibody-based CAR-T cell that demonstrated efficacy in various metastatic and orthotopic small cell lung cancer mouse models with medium (H82 and SHP-77) and low (H69) DLL3 surface expression levels. This approach can improve the anti-tumor efficacy of CAR-T cells, enhance their infiltration into tumors, and modulate the TME to activate immune cells.

Notch signaling plays a crucial role in regulating the activation, infiltration, and phenotypic switching of various immune cells. The gene circuit comprises three fundamental processes: input, circuit, and output. SynNotch receptors exemplify this gene circuit concept, as they can be engineered to customize the anti-tumor response of T cells. In scenarios where CAR signaling is partially suppressed by immunosuppressive TME conditions, the synNotch circuit can independently activate CAR signaling pathways, allowing for the sustained activation and proliferation of CAR-T cells in challenging environments.^19^ The synNotch-based antigen-dependent cytokine release strategy not only facilitates efficient expansion of CAR-T cells in immunosuppressed TMEs, but also significantly enhances the overall efficacy of armored CAR-T therapies against solid tumors. In a mouse lung cancer model that recapitulates the immunosuppressive TME of human patients, activating Notch signaling in myeloid cells promoted the differentiation of monocytic myeloid-derived suppressor cells into M1-type TAMs, which helped inhibit tumor progression. This highlights the multifaceted role of Notch signaling in shaping the immune landscape within the TME. Overall, the synNotch-CAR approach represents a promising advancement in CAR-T therapy, enhancing both specificity and efficacy against solid tumors while minimizing potential off-target effects.

CAR-T cell therapy in combination with traditional therapies has shown promising potential in treating solid tumors such as lung cancer. Chemotherapy can contribute to remodeling the TME, which may enhance the therapeutic effects of CAR-T cells in solid tumors. Srivastava et al^20^ found that receptor tyrosine kinase-like orphan receptor 1 (ROR1)-targeted CAR-T therapy was ineffective because of poor T cell infiltration; their study showed that oxaliplatin/cyclophosphamide (Ox/Cy) chemotherapy enhanced CAR-T cell infiltration in Kras^LSL-G12D/+^;p53^f/f^ lung adenocarcinoma and increased tumor sensitivity to anti-programmed cell death ligand 1 (PD-L1), improving CAR-T mediated tumor control and five-year survival rate. Clinical and preclinical studies indicate that certain chemotherapy drugs can enhance the transport, invasion, expansion, and anti-tumor efficacy of CAR-T cells against solid tumors by improving the TME. These effects, however, are closely related to the type and dosage of the chemotherapy used.

Radiation therapy (RT), a standard cancer treatment, employs high doses of radiation to target and destroy cancer cells. In addition to directly killing tumor cells, RT can activate the immune response within the TME, enhance antigen presentation, and promote the infiltration of CD8^+^ T cells and NK cells into tumors. While RT may damage CAR-T cells, clinical studies have shown that low-dose RT (2 Gy) can induce *in vivo* expansion of CAR-T cells.^21^ Quach et al^22^ integrated non-ablative tumor-targeted RT with CAR-T cell therapy in models of pleural mesothelioma and NSCLC, overcoming the barrier of poor T cell infiltration, increasing tumor-infiltrating CAR-T cells, and upregulating central memory phenotype and chemokine receptors, thus enhancing anti-tumor efficacy in solid tumors. In conclusion, the prospects for combination therapy strategies incorporating CAR-T cells are highly promising, particularly regarding their impact on the TME in solid tumors such as lung cancer. Further research is essential to optimize treatment dosages, sequencing, and other relevant factors.

Future research will need to focus on elucidating the mechanisms underlying the action of CAR-T cells in lung cancer and other solid tumors, as well as identifying more effective interventions. This will help broaden the application and facilitate the clinical translation of CAR-T cell therapy in solid tumor treatment. On April 13, 2025, C—CAR031, the world's first autologous glypican-3 (GPC3)-targeted CAR-T therapy, completed the first patient reinfusion in a Shanghai Clinical Medical Center for Lung Tumors study on advanced squamous cell lung cancer. A year prior, C—CAR031 showed promising results in advanced liver cancer patients with multi-line treatment failures, demonstrating manageable safety and strong anti-tumor activity. This latest success not only validates the therapy's technical viability but also offers a new treatment option for patients with immunotherapy resistance. With ongoing advancements in science and technology, coupled with deeper investigative efforts, we are optimistic that CAR-T cell therapy will achieve even greater success in treating solid tumors. Such progress will not only provide novel ideas and strategies for lung cancer treatment but also drive innovation and improvement in therapeutic approaches for patients suffering from other solid tumors.

## CRediT authorship contribution statement

**Qi Zhou:** Writing – review & editing. **Fengfei Sun:** Writing – review & editing. **Xinhui Wang:** Writing – review & editing, Supervision.

## Declaration of competing interest

The authors declare that they have no known competing financial interests or personal relationships that could have appeared to influence the work reported in this paper.
